# *TEF-7A*, a transcript elongation factor gene, influences yield-related traits in bread wheat (*Triticum aestivum* L.)

**DOI:** 10.1093/jxb/eru306

**Published:** 2014-07-23

**Authors:** Jun Zheng, Hong Liu, Yuquan Wang, Lanfen Wang, Xiaoping Chang, Ruilian Jing, Chenyang Hao, Xueyong Zhang

**Affiliations:** ^1^Crop Genomics and Bioinformatics Center and National Key Lab of Crop Genetics and Germplasm Enhancement, College of Agricultural Sciences, Nanjing Agricultural University, Nanjing 210095, Jiangsu, China; ^2^Key Laboratory of Crop Gene Resources and Germplasm Enhancment, Ministry of Agriculture/The National Key Facility for Crop Gene Resources and Genetic Improvement/Institute of Crop Science, Chinese Academy of Agricultural Sciences, Beijing 100081, China; ^3^Institute of Wheat Research, Shanxi Academy of Agricultural Sciences, Linfen 041000, China

**Keywords:** Association analysis, haplotypes, transcript elongation factor, wheat, yield.

## Abstract

In this study, *TaTEF-7A*, a member of the transcript elongation factor gene family, and its ﬂanking sequences were isolated. *TaTEF-7A* was located on chromosome 7A and was ﬂanked by markers *Xwmc83* and *XP3156.3*. Subcellular localization revealed that TaTEF-7A protein was localized in the nucleus. This gene was expressed in all organs, but the highest expression occurred in young spikes and developing seeds. Overexpression of *TaTEF-7A* in *Arabidopsis thaliana* produced pleiotropic effects on vegetative and reproductive development that enhanced grain length, silique number, and silique length. No diversity was found in the coding region of *TaTEF-7A*, but 16 single nucleotide polymorphisms and Indels were detected in the promoter regions of different cultivars. Markers based on sequence variations in the promoter regions (InDel-629 and InDel-604) were developed, and three haplotypes were identified based on those markers. Haplotype–trait association analysis of the Chinese wheat mini core collection revealed that *TaTEF-7A* was significantly associated with grain number per spike. Phenotyping of near-isogenic lines (NILs) confirmed that *TaTEF-7A* increases potential grain yield and yield-related traits. Frequency changes in favoured haplotypes gradually increased in cultivars released in China from the 1940s. Geographic distributions of favoured haplotypes were characterized in six major wheat production regions worldwide. The presence of *Hap-7A-3*, the favoured haplotype, showed a positive correlation with yield in a global set of breeding lines. These results suggest that *TaTEF-7A* is a functional regulatory factor for grain number per spike and provide a basis for marker-assisted selection.

## Summary

*TaTEF-7A* is a functional regulatory factor gene for grain number and other traits, and its diagnostic markers could be used in marker-assisted selection to improve yield potential in wheat.

## Introduction

Transcript elongation is a simple extension of an initiated transcript (Grasser, 2005). In recent years, it has become apparent that synthesis of mRNA is a dynamic and highly regulated step in gene expression, and it requires the concerted action of a variety of elongation factors (Lolas *et al.*, 2010). Transcript elongation factors (TEFs) play a crucial role in regulation, proliferation, and differentiation of cells, and also control various growth processes. The vast majority of studies on the regulation of eukaryotic transcription have focused on the early stages of the transcription cycle. However, studies of *Drosophila* and zebraﬁsh mutants defective in elongation factors revealed intriguing results about the essential roles of these factors in development. Mutations in the *Drosophila* gene encoding a member of the ELL family (*dELL*), which belongs to the TEF group, cause segmentation defects and embryonic lethality, and *dELL* is required for normal expression of diverse genes during development (Eissenberg *et al.*, 2002). Characterization of the effect of a *foggy* mutant on the TEFs Pandora/Spt6 and Foggy/Spt5 with an amino acid substitution in zebraﬁsh revealed that the mutation not only affected typical overall anatomy but also influenced the differentiation of cells in speciﬁc neuron lineages (Keegan *et al.*, 2002). Recent studies in *Arabidopsis* and maize also indicated that factors regulating transcript elongation play crucial roles in plant development. Mutations in the *Arabidopsis DEFORMED ROOTS AND LEAVES1* (*DRL1*) gene cause disorganized shoot, inﬂorescence, ﬂower, and root growth (Nelissen *et al.*, 2003). *DRL1* is likely to be involved in regulation of meristem activity and organ growth. Mutations in three genes encoding subunits of the *Arabidopsis* elongator complex display a pleiotropic phenotype, to some extent resembling the phenotype of the *drl1* mutant (Nelissen *et al.*, 2005). Thus, *elo* mutants show reduced growth of organs that can be attributed to a reduced rate of cell proliferation. Molecular analysis of *elo* mutants revealed that many genes were abnormally expressed in the mutants compared with the wild types (Nelissen *et al.*, 2005). A study of the maize *Etched* (*et1*) mutant revealed the intriguing possibility that control of transcript elongation also plays a crucial role in expression of plastid gens; *et1* mutants display delayed greening of seedling leaves. Mature kernels of these plants are severely ﬁssured and cracked. Microscopic examination demonstrated aberrant plastid development in mutant leaves and kernels (Da Costa e Silva *et al*., 2004).

In rice, a T-DNA insertion mutant in a gene encoding a TEF homologous to yeast *elf1* (*Os2TEF1*) led to a 60–80% reduction in tillering, retarded growth of seminal roots, and sensitivity to salt stress compared with wild-type Basmati 370. Detailed transcriptomic profiling of *OsTEF1* revealed that mutation in the TEF differentially regulated expression of >100 genes with known function and finally regulated the tillering process by inducing expression of cytochrome P450 (Paul *et al.*, 2012). These findings show that the TEFs are more pleiotropic than previously thought, and that previous studies failed to identify all functions of the factors.

Bread wheat (*Triticum aestivum* L.), as one of the three most important cereals, is widely cultivated worldwide, and improvement of wheat yields could prevent global food shortages. As it is an allohexaploid species with an extremely large and complex genome (2*n*=6*x*=42) of ~16 000 Mbp (Tilman *et al.*, 2011), gene isolation is extremely difficult. It is well known that grain yield is manifested via a complex relationship among yield component traits such as 1000-kernel weight (TKW), grain number per spike (GN), and effective tiller number (Zhang *et al.*, 2010). Thus, deciphering the molecular mechanisms underlying yield-related genes in wheat is of great interest not only to plant biologists, but also to breeders. Wheat yield, or yield components, is controlled by numerous genes with additive and epistatic effects that are highly interactive with the environment. Quantitative trait locus (QTL) mapping has been widely used to study specific yield-related traits in bread wheat (Zhang *et al.*, 2012). Numerous QTLs have been reported for traits such as TKW, GN, grain weight per spike, and tiller number (Huang *et al.*, 2004; Narasimhamoorthy *et al.*, 2006), but ﬁne mapping of particular genes from QTLs associated with yield is rarely reported (Barrero *et al.*, 2011). Fine mapping is hampered by the large genome and large number of repeated nucleotide sequences. A few genes associated with yield have been isolated by comparative genetics. For example, an orthologue of the rice *OsGW2* gene, named *TaGW2*, was cloned in wheat; this gene significantly affected TKW (Su *et al.*, 2011; Yang *et al.*, 2012). Zhang *et al.* (2012) characterized *TaCKX6-D1* that also increased grain productivity, and further analysis showed that it explained phenotypic variance of 1.3–1.4g per 1000 kernels. The function and mechanism of a glutamine synthetase gene controlling nitrogen use was comprehensively studied by Li *et al.* (2011) and Quraishi *et al.* (2011), who found that the best haplotype conferred superior seedling growth, better agronomic performance, and improved N uptake during vegetative growth and grain N concentration. Xue *et al.* (2011) identified *TaMYB13*, a transcriptional activator of fructosyltransferase genes, that is involved in β-2, 6-linked fructan synthesis in wheat, and positively associated with grain yield. Currently, there is an increasing need to clone yield-related genes, exploit the more favourable alleles, and develop tailor-made markers for marker-assisted selection (MAS).

In this study, work is presented on isolation of the gene *TaTEF-7A* in wheat and its functional characterization by expression analysis, association analysis, near-isogenic line (NIL) comparison, and overexpression in *Arabidopsis.* The ﬁndings indicate that *TaTEF-7A* mainly regulates yield-related traits involved in vegetative growth and reproductive development. Furthermore, the coding and promoter regions were analysed and sequence polymorphisms among wheat cultivars were identified. Association analysis and comparison of NILs showed that *TaTEF-7A* was associated with grain number. Finally, a functional marker for *TaTEF-7A* was developed for MAS of high-yielding wheat genotypes.

## Materials and methods

### Plant materials

A Chinese wheat mini core collection (MCC) was employed for association of yield traits with markers. The MCC contains 262 wheat accessions, comprising 157 landraces, 88 modern cultivars, and 17 introduced lines, and represents 1% of the national wheat collection but >70% of the genetic diversity (Hao *et al.*, 2011). Accessions of the three putative diploid progenitors of hexaploid common wheat (AABBDD), namely *Triticum urartu* (AA, accession UR203), *Aegilops speltoides* (SS, closely related to BB, accession Ae49), and *Aegilops tauschii* (DD, accession Y2282) were used for identiﬁcation of the genomic origins of *TaTEF* homoeologues. A set of nullisomic–tetrasomic lines of Chinese Spring was used for chromosomal location of *TaTEF* genes. A double haploid (DH) mapping population (Hanxuan 10×Lumai 14) was used for linkage mapping. NILs for *TaTEF*-7A were identified in a BC_3_F_6_ of Jinmai 47//4*/Lumai14 population. In addition, a large number of modern wheat cultivars, comprising 384 European, 429 North American, 53 CIMMYT, 82 Russian, and 51 Australian cultivars (Hou *et al.*, 2014), were used to determine the geographic distribution of *TaTEF-7A* haplotypes.

### Phenotypic assessment and statistical analyses

Phenotypic traits of all accessions in the MCC, namely heading date (HD), maturity date (MD), spike length (SL), spikelet number per spike (SN), plant height (PH), grain number per spike (GN), effective tiller number (ETN), 1000-kernel weight (TKW), kernel length (KL), kernel width (KW), and kernel thickness (KT), were collected in four environments—in 2002, 2005, and 2006 at Luoyang, Henan province, and in 2010 at Shunyi, Beijing; they were named 02LY, 05LY, 06LY, and 10SY, respectively. Each accession was planted in a 2 m two-row plot with 30cm between rows, and 40 seeds per row. Ten plants from the middle of each plot were used in investigating the above phenotypic traits. The DH mapping population (Hanxuan 10×Lumai 14) and NILs were planted at Changping, Beijing. Five plants in the middle of each plot were randomly sampled for analysis. Eight agronomic traits were measured, namely PH, SL, TKW, GN, ETN, SN, penultimate internode length, and uppermost internode length.

Mean values of phenotypic traits and standard errors were analysed by SPSS 16.0 software (http://www.brothersoft.com/downloads/spss-16.html). The mean value of each trait in the Chinese wheat MCC was estimated by the best linear unbiased predictor (BLUP) method (Bernardo *et al*., 1996*a*, *b*, *c*).

### DNA and RNA extraction

Genomic DNA was extracted from young leaves of each accession using the cetyltrimethylammonium bromide (CTAB) method (Stewart and Via, 1993). Total RNA was isolated using an RNAprep pure plant kit (Tiangen) according to the manufacturer’s instructions. cDNA was synthesized with M-MLV Reverse Transcriptase (Promega), the cDNA was diluted 10 times, and 2 μl were used for subsequent PCR.

### Primers and PCR

Primers were designed by the software Primer Premier Version 5.0 (Premier Biosoft International, Palo Alto, CA, USA), and the primers were synthesized by Sangon (www.sangon.com). The Primer sequences are shown in Supplementary Table S1 available at *JXB* online. LA-Taq enzyme from TaKaRa (www.takara.com.cn) was used for PCR ampliﬁcation. PCR were performed in total volumes of 15 μl, including 3 pmol of each primer, 120 μM of each dNTP, 80ng of template DNA or cDNA, 0.75U of La-Taq, and 7.5 μl of 2× buffer [TaKaRa Biotechnology (Dalian) Co. Ltd, Product Code: DRR20AG]. PCRs were performed as follows: 95 °C for 4min; followed by 30–35 cycles of 95 °C for 30 s, annealing (55–62 °C) for 30 s, and extension at 72 °C (30 s to 3min), and 72 °C for 30 s, with a final extension of 72 °C for 10min. The annealing temperatures and extension times depended on the primer sets and lengths of expected PCR products.

### Functional marker development

Development of TaTEF-4F/R was based on two selected variations in the promoter region of *TaTEF-7A*, including a 1bp indel at position –629bp (InDel1-629) and another 1bp indel at position –604bp (InDel1-604). First, genomic-speciﬁc primer set TaTEF-3F/R was used to amplify fragments from chromosome 7A in all cultivars. A second PCR was performed as follows: the first PCR solution was diluted 100 times, taking 1 μl as template for the second PCR, primer TaTEF-4F/R was used for the second PCR, with annealing at 54 °C for 30 s, and extension at 72 °C for 30 s.

### Chromosomal localization

To determine the chromosomal location, 37 nulli-tetrasomic lines of Chinese Spring provided by the Kansas Wheat Stock Centre (http://www.k-state.edu/wgrc/) were used. Gene-specific primers TaTEF-1F/R, TaTEF-2F/R, TaTEF-3F/R, and TaTEF-6F/R for *TaTEF*-*7A* localization were used for PCR. *TaTEF-7A* was mapped on a mapping population (Hanxuan 10×Lumai 14) using the MAPMAKER/EXP 3.0 (Lander and Botstein, 1989).

### Multiple sequence alignment and phylogenetic analysis

Full-length protein sequences of TaTEF-7A from *Triticum aestivum* (*Ta*), *Brachypodium distachyon* (*Bd*), *Oryza sativa* (*Os*), *Setaria italica* (*Si*), *Zea mays* (*Zm*), *Sorghum bicolour* (*Sb*), *Arabidopsis thaliana* (*At*), *Thellungiella halophila* (*Th*), *Brassica rapa* (*Br*), *Citrus sinensis* (*Cs*), *Physcomitrella patens* (*Pp*), and *Saccharomyces cerevisiae* (*By*) were aligned by ClustalX. A phylogenetic tree was created using a Neighbor–Joining method in the MEGA5.05 program (Tamura *et al.*, 2011).

### Subcellular location of TaTEF-7A protein by fusion with green ﬂuorescent protein (GFP)

The full-length cDNA clone of *TaTEF-7A* was fused upstream of the GFP gene and put under control of the constitutive *Cauliflower mosiac virus* (CaMV) 35S promoter in the pJIT163-GFP expression vector to construct a 35S::TaTEF–GFP fusion protein. Restriction sites, *Kpn*I and *Xba*I, were added to the 5’ and 3’ ends of the coding region by PCR. The PCR products were digested with restriction endonucleases, ligated with the pJIT163-GFP plasmid, and cut with the corresponding enzymes to create recombinant plasmids expressing the fusion protein. The subcellular location of TaTEF-7A was detected by monitoring transient expression of GFP in wheat protoplast cells as described by Mao *et al.* (2011)

### Expression analysis

Quantitative real-time PCR was performed using SYBR^®^ Premix Ex Taq™ II (Takara) according to the manufacturer’s instructions on a 7300 Real-time PCR System (Applied Biosystems). The relative expression of each gene was calculated according to the 2^–ΔΔCT^ method (Livak and Schmittgen, 2001). The glyceraldehyde-3-phosphate dehydrogenase gene was used as an endogenous reference for real-time PCR, and all analyses were performed with three technical and three biological replicates.

### Binary vector construction and transformation of *Arabidopsis*

The coding sequence of *TaTEF-7A*, including the *Xba*I (5′) and *Bam*HI (3’) restriction sites, was first amplified. The PCR products and pbi121 plasmid were digested with *Xba*I and *Bam*HI, and then cloned into the binary vector pbi121 under control of the CaMV 35S promoter in the sense orientation. The construct was transformed into *Arabidopsis* (ecotype Col-0) by the floral-dip method according to previously described procedures using *Agrobacterium tumefaciens* strain GV3101 (Herrera-Estrella *et al.*, 2005). Transgenic plants were selected on solid half-strength Murashige and Skoog (MS) medium containing 50mg l^–1^ kanamycin (Ahmed *et al.*, 2012).

### Transient expression in tobacco

The promoter fragment of *TaTEF-7A* was fused with the *β-glucuronidase* (*GUS*) gene in the pCAMBIA1391Z vector. Restriction sites, *Bam*HI and *Avr*II, were added to the 5′ and 3′ ends of the coding region by PCR. The PCR products were digested with restriction endonucleases, and then ligated with the plasmid. pBICHP57. The luciferase (LUC) reporter gene (Wu *et al.*, 2009) was cloned in the binary vector pBI121 downstream of the CaMV 35S promoter. *Nicotiana benthamiana* leaves were co-infiltrated with plasmids and pBICHP57. The *GUS* activity was normalized by the *LUC* activity. Plant transformation, and *GUS* and *LUC* activity assays were determined according to Wu *et al.* (2009).

### Plant growth and documentation

*Arabidopsis thaliana* (ecotype Columbia-0) was chosen for transgene analysis, and the T_3_ generation was grown in a controlled environment chamber at 22 °C, with a 16h/8h photoperiod, light intensity of 120 mmol m^−2^ s^−1^, and 70% relative humidity. After sowing, seeds were stratiﬁed in darkness at 4 °C for 48h prior to incubation in a plant growth chamber. At least five plants of each line were used for phenotypic characterization. Distances from the stem bases in each line were measured to compare silique lengths. The numbers of siliques on each line were counted. Seeds of each line were photographed and measured using an Olympus BH-2 microscope (Olympus, Japan).

## Results

### Isolation of *TaTEF-7A* and phylogenetic analysis of orthologous genes in wheat and other species

Using the rice *TEF* gene (Os02g04160) as a query, contig08999 was found in the Chinese Spring draft genome sequence (http://www.cerealsdb.uk.net/cerealgenomics/CerealsDB/Documents/DOC_search_reads.php). A full-length wheat coding sequence (CDS; accession CJ655632) was retrieved from NCBI by blasting the contig sequence against GenBank (Supplementary Fig. S1 available at *JXB* online). The *TaTEF* genomic sequence and CDS were ampliﬁed by TaTEF-5F/R from Chinese Spring genomic DNA and cDNA, respectively. Their lengths were ~1100bp and 270bp, respectively. cDNA sequences of the three homoeologous genes in the A, B, and D genomes were obtained (Supplementary Figs S2, S3 available at *JXB* online). Four exons and three introns were detected in this gene (Supplementary Fig. S1 available at *JXB* online).

*TaTEF* encodes an 89 amino acid polypeptide that is 84.9% identical to OsTEF protein (Supplementary Fig. S1b available at *JXB* online). In most species, *TEF* genes have similar structures with four exons and three introns. A phylogenetic tree of *TEF* genes was generated based on the predicted polypeptide sequences in monocots, eudicots, and yeast (Supplementary Table S2 available at *XB* online). All of the proteins shared high similarity of 52.8–95.5%, implying potentially conserved functions among orthologues (Supplementary Fig. S1c available at *JXB* online). The tree consists of at least three divergent clades with strong bootstrap support; the species include monocots, eudicots, and *Saccharomyces cerevisiae*.

### Diversity mainly occurs in the promoter region of *TaTEF* on chromosome 7A

**Fig. 1.**
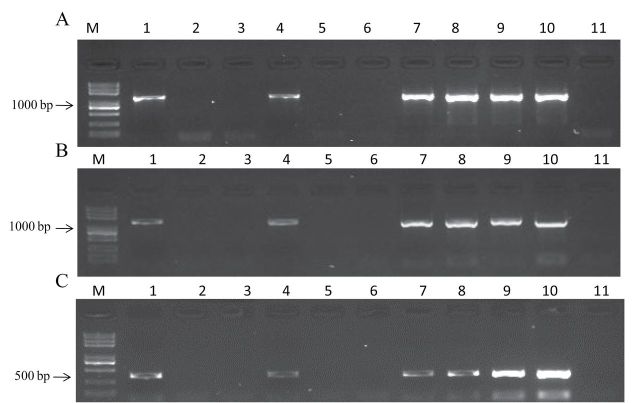
Targeted PCR ampliﬁcation of *TaTEF*-7A in *T. aestivum* cv. Chinese Spring, the nullisomic–tetrasomic lines for homoeologous group 7, and three putative diploid progenitors of wheat with genome-speciﬁc primer sets. (A) Primer TaTEF-1F/R; (B) primer TaTEF-2F/R; (C) primer TaTEF-3F/R. M, marker; 1, *T. urartu*; 2, *Ae. speltoides;* 3, *Ae. tauschii*; 4, *T. aestivum*; 5, N7AT7B; 6, N7AT7D; 7, N7BT7A; 8, N7BT7D; 9, N7DT7B; 10, N7DT7A; 11, H_2_O.

**Fig. 2.**
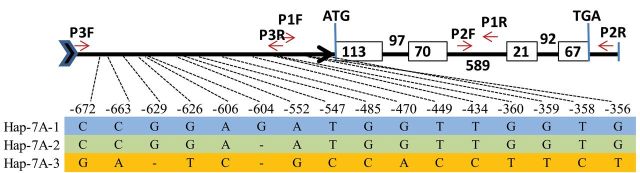
Gene structure and haplotypes in the promoter region of *TaTEF-7A*. The positions of 14 SNPs and two Indels between cultivars are shown using the *TaTEF* gene sequence, with the start codon designated as position 0. Haplotypes are highlighted in different colours. Arrow bar, promoter region; open boxes, exons; thin lines, introns; ATG, start codon; TGA, stop codon; red arrows, positions of primers TaTEF-1F/R, TaTEF-2F/R, and TaTEF-1F/R.

A number of single nucleotide polymorphisms (SNPs) and Indels were found in the intron sequences of *TaTEF* genes from the A, B, and D genomes. This allowed discrimination of *TaTEF-7A* from the other copies on chromosomes 7B and 7D by amplification of genome-specific primers TaTEF-1F/R, TaTEF-2F/R, and TaTEF-3F/R (Fig. 1). The region of each primer set is shown in Fig. 2. No nucleotide difference was detected in the coding region from 48 cultivars with quite different phenotypes, implying that the mechanism of control of wheat development possibly depends on regions external to the gene. Approximately 700bp upstream of ATG of the 48 accessions were then sequenced. Fourteen nucleotide substitutions and two Indels were detected among the 48 cultivars, and three major haplotypes were formed by 16 representative nucleotides (Fig. 2; Supplementary Table S3 available at *JXB* online).

**Fig. 3.**
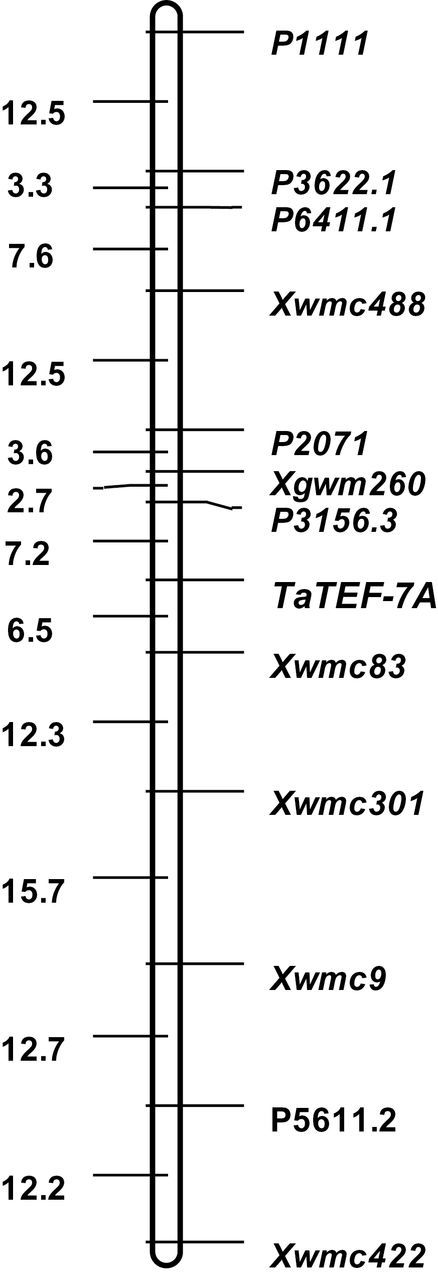
Linkage map of *TaTEF-7A* and surrounding markers on wheat chromosome 7A.

The design of primer TaTEF-3F/3R was based on polymorphisms in the promoter region. As shown in Fig. 1C, target fragments were detected only in accessions with the A genome, and were not amplified in *Ae. speltoides, Ae. tauschii*, NT7A7B, and NT7A7D. This indicated that *TaTEF* was amplified from chromosome 7A in *Triticum* spp. Moreover, primer TaTEF-4F/R (Table 1) was used to genotype 150 DHs derived from Hanyuan10×Lumai14. *TaTEF* was mapped to a region ﬂanked by *P3156.3* (7.2 cM distal) and *Xwmc83* (6.5 cM proximal) on chromosome 7A (Fig. 3).

**Table 1.** Phenotypic analysis of Col-0 and two transgenic plants

Trait	CK	Line 1	Line 2
Silique length (cm)	1.06±0.05 a(A)	1.23±0.06 b(B)	1.13±0.069 c(B)
Silique number	21.0±3.08 a(A)	31.5±2.87 b(B)	27.5±2.41 c(C)
Grain length (μm)	441.26±31.37 a(A)	489.03±7.85 b(B)	498.99±14.3 6 b(B)

CK, Col-0.

Upper and lower case letters indicate significance level at *P*=0.01 and *P*=0.05, respectively.

### *TaTEF-7A* is mainly expressed in young spikes and developing seeds, and the protein is located in the nucleus

**Fig. 4.**
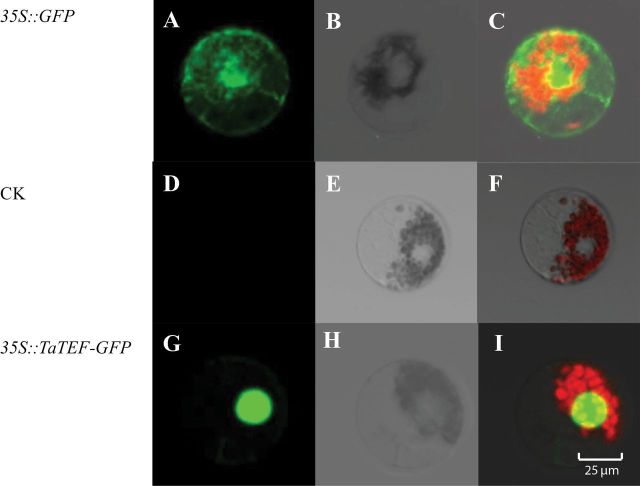
Subcellular localization of TaTEF-7A protein in the wheat protoplast. Cell images of green fluorescence in dark field (A, D, G); cell images in bright field (B, E, H); merged images (C, F and I). CK, control.

**Fig. 5.**
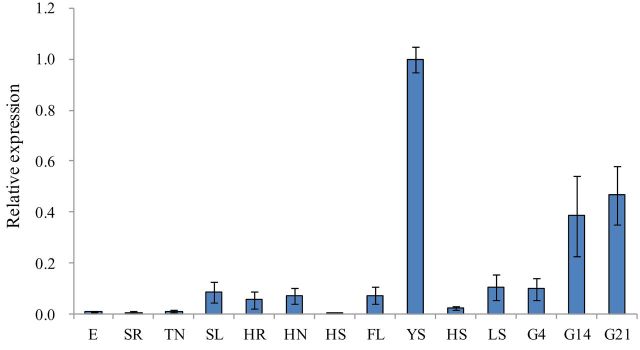
*TaTEF-7A* expression levels revealed by real-time PCR amplification. E, embryo; SR, seedling root; TN, tillering node; SL, seedling leaf; HR, roots at heading date; HN, nodes at heading date; HS, stems at heading date; FL, flag leaves; YS, young spike 1.5–2.5cm in length; HS, spike 6–7 in cm length; LS, leaf sheath; G4, grain 1–4 DAF; G14, grain 13–14 DAF; G21, grain 20–21 DAF; the expression level of young spikes 1.5–2.5cm in length was assumed to be 1.00 and was regarded as the reference for other tissues.

Transient expression of *TaTEF-7A* fused with *GFP* in wheat protoplast cells showed that *TaTEF7A-GFP* accumulated only in the nucleus, whereas GFP alone was present throughout the whole cell (Fig. 4), demonstrating that *TaTEF-7A* interacted with the cell nucleus. TaTEF-6F/R (Table 1) was designed based on the 5’-untranslated region (UTR) sequence, which was amplified only from the 7A chromosome (Supplementary Fig. S4 available at *JXB* online). A tissue-specific expression study using quantitative real-time PCR showed that *TaTEF-7A* was expressed in nearly all tissues tested (Fig. 5). The highest expression levels were detected in young spikes and developing seeds. The transcription level was consistent in spikes at various stages from young to immature seeds and increased gradually during grain filling, indicating that it may have effects on yield traits.

### Overexpression of *TaTEF-7A* in *Arabidopsis* alters vegetative and reproductive development

**Fig. 6.**
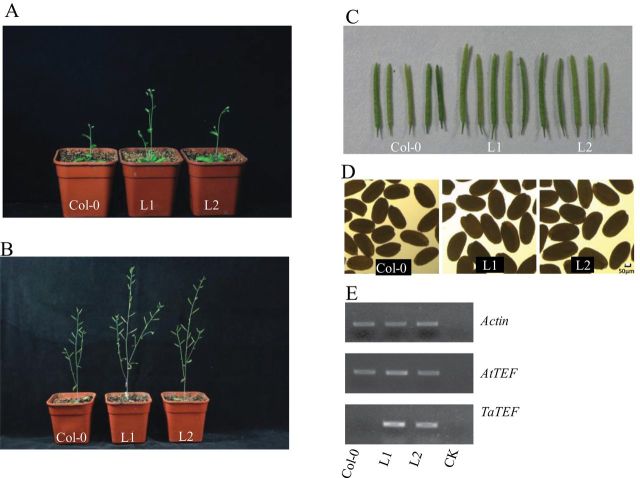
Overexpression of *TaTEF-7A* in *Arabidopsis*. Comparisons of two trangenic lines and control plants: (A) growth at 23 days after sowing (DAS); (B) growth at 45 DAS; (C) siliques; (D) seeds; (E) *TaTEF-7A* expression.

Tissue-specific expression showed that *TaTEF-7A* was constitutively expressed, but at much higher levels in young spikes and developing seeds. A T-DNA insertion in rice caused 60–80% less tillering, retarded growth of seminal roots, and increased sensitivity to salt stress compared with the wild type (Paul *et al.*, 2012). The question that arises is what traits are affected when *TaTEF-7A* is overexpressed in *Arabidopsis*. Transgenic plants harbouring *TaTEF-7A* constructs driven by the CaMV 35S promoter were generated. Overexpression of *TaTEF-7A* in *Arabidopsis* enhanced vegetative growth of both seedlings and booting stage plants (Fig. 6A, B). As shown in Fig. 6C and D, and Table 1, silique and grain lengths were increased in transgenic lines 1 and 2 compared with the control. The expression levels of *TaTEF-7A* transgenics were also higher (Fig. 6E). The fact that *TaTEF-7A* affects silique length, silique number, and grain length suggests that this gene should also influence yield traits in wheat.

### Haplotype association indicates that *TaTEF-7A* strongly influences yield traits

**Fig. 7.**
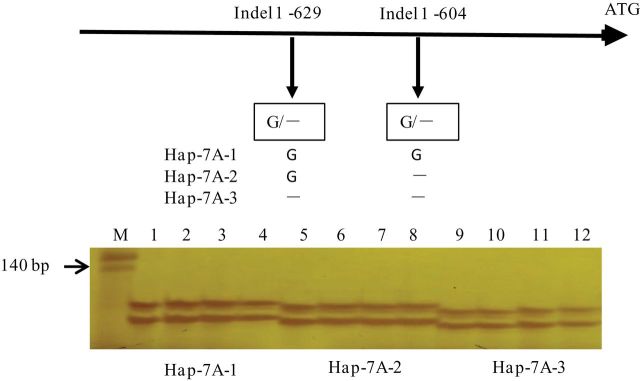
Marker development and polymorphism of different cultivars on 6% denaturing polyacrylamide gels. M, marker; 1, LM14; 2, QCM; 3, WSB; 4, NQ4; 5, NY188; 6, BN6; 7, AM6; 8, PY27; 9, SJZ8; 10, YZ1; 11, S4185; 12, YM18.

Compared with *Hap-7A-1*, *Hap-7A-2* has a 1bp deletion and *Hap-7A-3* has a 2bp deletion. After amplification, the PCR products were separated using 6% denaturing polyacrylamide gels (Fig. 7). Linkage disequilibrium (LD) was estimated between all pairs of polymorphic sites in the 0.7kb region. Significant LD (*r* ^2^=1, *P*<0.001) was observed across pairwise polymorphisms (Supplementary Fig. S5 available at *JXB* online).

The MCC is regarded as a suitable population for detection of major QTLs controlling yield traits (Zhang *et al.*, 2012). In comparing the effects of each haplotype on agronomic traits GN, KL, KW, KT, and TKW in MCC landraces, significant associations were identified, and there was a negative relationship between GN and TKW. However, among modern cultivars, there were significant differences in GN between haplotypes. Interestingly, the phenotypic means for KL, KW, KT, and TKW in *Hap-7A-3* accessions were all higher than for other haplotypes in this subset, and GN differences between the three haplotypes were also significant. Thus the average GN of *Hap-7A-3* was increased, with TKW enhanced (*P*<0.05) between landraces and modern cultivars during yield improvement (Table 2). Collectively, these results demonstrated that *Hap-7A-3* was positively selected because it had a significantly positive effect on yield.

**Table 2.** Comparisons of three haplotypes of *TaTEF-7A* based on BLUP values of phenotypic traits in MCC from four environments

Genotype/trait	Hap-7A-1		Hap-7A-2		Hap-7A-3	
	Mean ±SE	Range	Mean± SE	Range	Mean ±SE	Range
Landraces						
HD (d)	196.86±0.42 a	188.00–209.00	197.78±0.98 a	194.00–202.00	192.33±2.96 a	188.00–198.00
MD (d)	237.35±0.34 a	230.00–248.00	237.56±0.69 a	235.00–241.00	234.00±1.53 a	232.00–237.00
SL (cm)	9.89±0.14 a	6.34–14.07	10.40±0.60 a	7.40–12.69	11.44±0.55 a	10.40–12.27
SN	21.65±0.09 a	18.93–27.64	20.94±0.44 a	19.03–22.78	20.78±0.35 a	20.28–21.46
PH (cm)	113.54±0.82 a	58.96–145.23	116.15±1.91 a	109.77–128.73	113.96±5.32 a	108.21–124.58
GN	49.73±0.46 a	38.29–72.92	45.07±1.84 b	37.79–52.80	43.37±3.55 a	38.69–50.34
ETN	10.68±0.10 a	7.83–14.05	10.84±0.40 a	8.65–12.73	10.10±1.21 a	8.59–12.49
TKW (g)	32.04±0.38 A	22.77–46.11	35.48±1.26 A	28.30–39.95	46.48±3.83 B	39.59–52.81
KL (mm)	6.35±0.04 A	5.55–7.45	6.62±0.15 A	6.18–7.37	7.70±0.26 B	7.18–8.02
KW (mm)	3.01±0.01 a	2.60–3.40	3.07±0.04 ab	2.87–3.27	3.22±0.02 b	3.20–3.26
KT (mm)	2.75±0.01 a	2.35–3.16	2.85±0.05 ab	2.63–3.09	2.96±0.09 b	2.85–3.13
Modern cultivars						
HD (d)	194.00±0.47 a	189.00–208.00	195.00±1.52 a	190.00–201.00	192.25±0.65 a	190.00–198.00
MD (d)	235.65±0.42 a	228.00–245.00	236.63±1.13 a	234.00–243.00	234.25±0.95 a	230.00–242.00
SL (cm)	9.95±0.17 a	6.88–13.31	10.07±0.81 a	7.77–14.97	9.99±0.50 a	7.67–13.27
SN	21.38±0.12 a	19.40–23.88	21.34±0.49 a	19.69–23.85	21.49±0.23 a	20.13–22.56
PH (cm)	99.52±1.91 a	60.76–122.50	98.80±3.01 a	85.24–110.30	90.53±4.37 a	67.32–119.04
GN	50.31±0.68 a	37.40–63.97	48.44±1.89 a	42.76–58.07	55.39±1.87 b	43.85–65.35
ETN	9.99±0.18 a	7.15–13.96	10.41±0.38 a	9.30–12.15	9.03±0.24 a	7.94–10.26
TKW (g)	39.51±0.69 a	26.64–50.08	39.14±1.54 a	35.12–47.51	42.74±1.03 a	38.03–47.79
KL (mm)	6.71±0.06 a	5.75–7.85	6.57±0.10 a	6.18–7.04	6.89±0.08 a	6.36–7.26
KW (mm)	3.22±0.02 a	2.92–3.51	3.17±0.05 a	3.00–3.35	3.28±0.03 a	3.07–3.44
KT (mm)	2.91±0.02 a	2.62–3.24	2.91±0.05 a	2.66–3.19	2.94±0.04 a	2.72–3.18

Landraces: Hap-7A-1, *n*=145; Hap-7A-2, *n*=9; Hap-7A-3, *n*=3. Modern cultivars: Hap-7A-1, *n*=68; Hap-7A-2, *n*=8; Hap-7A-3, *n*=12

Upper and lower case letters indicate significant differences at *P*=0.01 and *P*=0.05, respectively.

Previous studies demonstrated that GN differences were associated with *TaELF-7A* haplotypes (Table 2). Therefore, differences in GN between *TaELF-7A* haplotypes under different environmental conditions were examined (Table 3). In landraces, the GN of *Hap-7A-1* was higher than those of *Hap-7A-2* and *Hap-7A-3* in all environments, but a significant difference (*P*<0.05) occurred only in 02LY. In modern cultivars, in contrast, the GN of *Hap-7A-3* was significantly higher than those of *Hap-7A-1* and *Hap-7A-2* in both 02LY and 10SY. Because *Hap-7A-3* had a signiﬁcantly positive effect on GN, it was considered a potentially superior allele for improvement of grain yield in wheat.

**Table 3.** Comparisons of *TaTEF-7A* haplotypes in MCC for GN in four environments

Haplotype	02LY		05LY		06LY		10SY	
	No.	Mean ±SE	No.	Mean ±SE	No.	Mean ±SE	No.	Mean ±SE
Landraces								
Hap-7A-1	129	51.00±0.99 a	129	40.70±0.63 a	145	54.63±0.79 a	142	52.70±0.76 a
Hap-7A-2	8	39.88±4.17 b	8	40.43±3.20 a	9	47.98±2.73 a	9	46.41±3.09 a
Hap-7A-3	3	37.00±2.65 ab	3	37.47±5.76 a	3	45.67±5.53 a	3	46.20±4.70 a
Modern cultivars								
Hap-7A-1	61	50.64±1.47 a	63	46.05±1.11 a	68	53.49±1.16 a	68	52.07±0.99 a(A)
Hap-7A-2	8	49.25±2.86 ab	8	42.05±1.08 a	8	50.08±3.94 a	8	50.90±2.92 a(AB)
Hap-7A-3	10	59.20±2.43 b	12	49.98±2.86 a	12	59.38±4.19 a	11	60.67±2.74 b(B)

02LY, Luoyang (2002); 05LY, Luoyang (2005); 06LY, Luoyang (2006); 10SY, Shunyi (2010)

Upper and lower case letters indicate significant differences at *P*=0.01 and *P*=0.05, respectively.

### The effects of *TaTEF-7A* haplotypes on yield traits were further confirmed in NIL comparisons

**Fig. 8.**
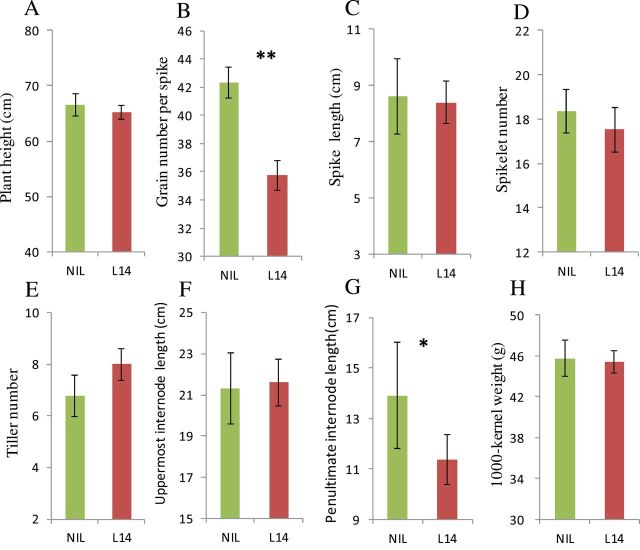
Phenotypic comparison of Lumai 14 (*Hap-7A-2*) and NIL (*Hap-7A-3*). (A) Plant height; (B) grain number per spike; (C) spike length; (D) spikelet number; (E) tiller number; (F) uppermost internode length; (G) penultimate internode length; (H) 1000-kernel weight; *P<0.05; **P<0.01.

NILs derived from a BC_3_F_6_ population of Jinmai 47//4*/Lumai14 were used to evaluate haplotype genetic effects, in which Jinmai 47 represented *Hap-7A-3* and Lumai 14 represented *Hap-7A-2*. Functional analysis of *TaTEF-7A* in NILs is shown in Fig. 8. It was clear that *Hap-7A-3* has higher GN (18.4%) than *Hap-7A-2*. Moreover, although *Hap-7A-3* (NIL) has a longer penultimate internode than *Hap-7A-2* (L14) (13.9±2.6cm versus 11.4±1.3cm), there was no significant difference in PH. *Hap-7A-3* also exhibited an increase in SN and TKW but a lower TN that did not reach statistical significance. This further confirmed that *Hap-7A-3* potentially increases grain yield through regulating grain number per spike.

### *TaTEF-7A* positively regulates yield-related traits

**Fig. 9.**
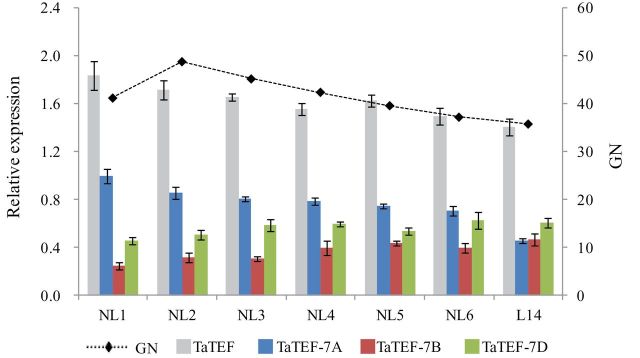
Relative expression levels of *TaTEF-7A*, *TaTEF-7B*, and *TaTEF-7D* in a set of NILs, and their relationship to GN. The expression level of *TaTEF-7A* was regarded as the reference and assumed to be 1.00.

**Fig. 10.**
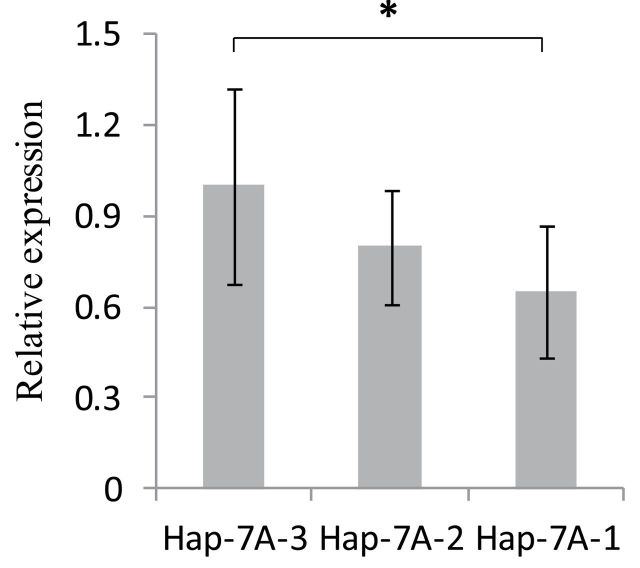
Quantification of the expression of *GUS* gene driven by each of the three haplotype promoters.

To confirm the expression patterns of *TaTEF-7A*, young spikes of each haplotype represented by four, one, and four varieties, respectively, were collected. Their relative expression values were measured by quantitative real-time qPCR (Table 4). *Hap-7A-3* was the highest overall, and a significant positive correlation between the expression level of *TaTEF-7A* and GN was observed (*r*=0.889, *P*<0.001) The average expression level of *TaTEF-7A* was significantly higher than that of either *TaTEF-7B* or *TaTEF-7D* in a set of NILs. GN was positively correlated with the total expression level of the three *TaTEF* genes. Furthermore, *TaTEF-7A* was the highest among the three homoeologues, except in the recurrent parent Lumai 14 (Fig. 9). Association analysis also showed that *Hap-7A-3* at *TaTEF-7A* was significantly related to higher GN, whereas *Hap-7A-1* and *Hap-7A-2* were associated with lower GN (Table 2). Transient expression analysis in tobacco also showed that the average relative expression of *Hap-7A-3* was higher than that for the other two haplotypes (Fig. 10). All of these results further demonstrated that *TaTEF-7A* positively regulated yield-related traits through its expression level.

**Table 4.** Difference in relative expression levels in different wheat varieties

Cultivar	Haplotype	Relative expression level of *TaTEF-7A*	GN
YM18	Hap-7A-3	0.53±0.03	58.6
WM6	Hap-7A-3	0.39±0.04	44.3
JM47	Hap-7A-3	0.36±0.03	48.4
S229	Hap-7A-3	0.32±0.02	47.8
PY27	Hap-7A-2	0.21±0.02	43.6
LM14	Hap-7A-2	0.14±0.01	39.5
ZY9507	Hap-7A-1	0.14±0.01	40.7
LZ953	Hap-7A-1	0.18±0.02	38.4
CS	Hap-7A-1	0.11±0.01	41.8

These cultivars were planted at Shunyi, Beijing.

The expression level of YM18 was assumed to be 1.00.

### *TaTEF-7A* co-locates with yield QTLs in wheat

*TaTEF-7A* was mapped using a DH population derived from Hanxuan 10×Lumai 14, and was flanked by SSR markers *P3156.3* (7.2 cM distal) and *Xwmc83* (6.5 cM proximal). This region of *TaTEF-7A* coincides with several previously reported yield-related QTLs, including spikelet number per spike *QSpn.nau-7A* (Ma *et al.*, 2007; Zhang *et al.*, 2010), flour yield *QFy550.b22-7A* (Kunert *et al.*, 2007), test weight *QTw.crc-7A*, and grain yield *QYld.crc-7A* (Huang *et al.*, 2006). In an immortalized F_2_ population of 136 lines derived from Nanda 2419×Wangshuibai, *QSpn.nau-7A* explained 10–12% of the spikelet variation (Ma *et al.*, 2007). Agronomic and quality traits were also detected in this region in a DH population from AC Karma’×87E03-S2B1. *QTw.crc-7A* for test weight and grain yield per plot explained 10.6% of the phenotypic variance in test weight, and *QYld.crc-7A* explained 8.1% of the variance in grain yield (Huang *et al.*, 2006). This locus was also related to flour yield in another study (Kunert *et al.*, 2007). An advanced backcross QTL (AB-QTL) strategy utilized to locate *QFy550.b22-7A* for baking quality traits in two BC_2_F_3_ populations of winter wheat explained 11.9% of the genetic variance (Kunert *et al.*, 2007). These results suggest that *TaTEF-7A* might be related to major yield QTLs mapped to the same region, and that the gene possibly confers complex pleiotropic effects on growth, yield, and quality.

### *Hap-7A-3* was positively selected in Chinese wheat breeding programmes

**Fig. 11.**
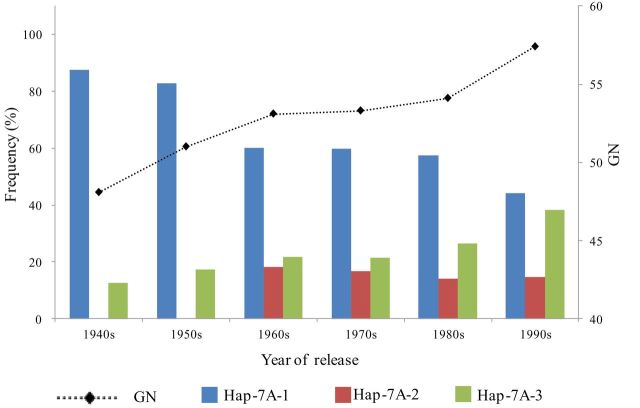
*TaTEF-7A* haplotype frequencies and GN/spike changes over decades in Chinese modern wheat cultivars released during the 1940s to 1990s.

A total of 348 modern cultivars released in China since the 1940s (Hao *et al.*, 2011) were classified into six subgroups (1940s, 1950s, 1960s, 1970s, 1980s, and 1990s) according to decade of release, and were used to determine haplotype frequencies of *TaTEF-7A* over time. The frequency of *Hap-7A-3* increased from 12.5% in the 1940s to 38.2% in the 1990s. Interestingly, the frequencies of this haplotype from the 1940s to 1990s were consistent with yield-related differences in GN. This strongly indicates that *Hap-7A-3* was positively selected in Chinese wheat breeding and that its frequency should be closely correlated with increases in GN (Fig. 11).

### Geographic distribution of haplotypes of *TaTEF-7A* in global wheat cultivars

**Fig. 12.**
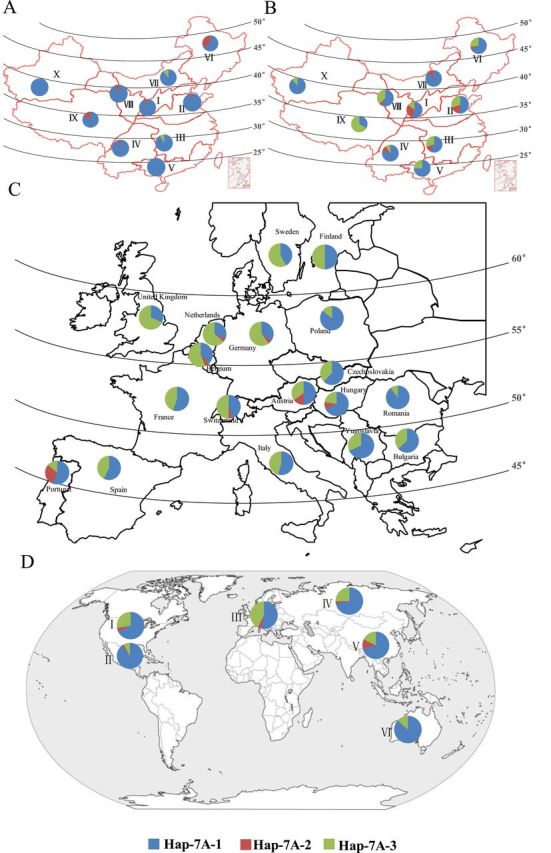
Global distribution of *TaTEF-7A* haplotypes. Distribution of 157 Chinese landraces (A) and 348 modern cultivars (B) in 10 production zones. I, northern winter wheat region; II, Yellow and Huai River valley winter wheat region; III, low and middle Yangtze River valley winter wheat region; IV, southwestern winter wheat region; V, southern winter wheat region; VI, northeastern spring wheat region; VII, northern spring wheat region; VIII, northwestern spring wheat region; IX, Qinghai–Tibet spring–winter wheat region; X, Xinjiang winter–spring wheat region. (C) Geographic distribution of haplotypes among European wheat cultivars; (D) geographic distribution of haplotypes in cultivars in other regions, I, North America; II, CIMMYT; III, Europe; IV, Former USSR; V, China; VI, Australia.

In order to evaluate comprehensively and systematically the distribution of all *TaTEF-7A* haplotypes in global wheat cultivars, haplotypes were determined in cultivars from North America, Australia, China, CIMMYT, Europe, and Russia. In Chinese landraces, *Hap-7A-1* was predominant across all zones (Fig. 12A), whereas the other two haplotypes had lower frequencies in some ecological regions. The favoured haplotype *Hap-7A-3* occurred only in two zones (III at a frequency of 8.7% and VII at 11.1%). A population with 348 Chinese modern cultivars was employed to reveal further the haplotype distributions in China. As shown in Fig. 12B, *Hap-7A-1* frequency declined in almost all zones except in VII. Interestingly, *Hap-7A-3* dramatically increased in different zones, particularly I (14.3%), II (29.9%), and III (29.4%), which have the oldest and strongest breeding programmes in China. It can be inferred that *Hap-7A-3* is the most favoured haplotype in breeding, because modern breeding has significantly promoted its frequency in Chinese modern cultivars (Jiang *et al.*, 2011; Su *et al.*, 2011).

In addition, haplotype analysis of 384 wheat cultivars released in 18 European countries showed that the average frequency of *Hap-7A-3* was 41.4%, ranging from 10% to 68.2%, which was much higher than that in cultivars released in China (Fig. 12C). *Hap-7A-3* distribution showed a geographic bias, with a higher frequency in western than in eastern Europe (Fig. 12C). Comparing the global distribution in six major international wheat regions (Fig. 12D), *Hap-7A-3* occurred at the highest frequency in varieties released in Russia and North America, but at lower frequencies in CIMMYT and Australian cultivars. This further demonstrated that the favoured haplotype *Hap-7A-3* underwent selective pressure but with different degrees in global wheat breeding.

## Discussion

### Genome rearrangement of the *TEF* orthologues occurred in grass crop evolution

*Poaceae* species have diverged >65 million years. Comparative mapping studies indicate that there is high synteny between species at the macro level (Sorrells *et al.*, 2003). An 89 amino acid wheat protein encoding the *TaTEF-7A* gene was identified using genome-specific primers and Chinese Spring nullisomic–tetrasomic lines. In rice, LOC_Os02g04160, the orthologue of *TaTEF-7A*, is located on chromosome 2. Collinearity was shown between wheat homoeologous group 6 and rice chromosome 2 (Bolot *et al.*, 2009; Su *et al.*, 2011). To date, there is little information to identify and characterize shared duplications within wheat and between rice and wheat. Interestingly, one model proposed a pathway for evolution of the rice, wheat, sorghum, and maize genomes from a common ancient ancestor (*n*=5) followed by genome duplication, with breakage and fusion of different chromosomes in the genomes of each of the four species (Salse *et al.*, 2008). Translocations and fusions were found between ancestral chromosomes A2, A4, and A6, which inevitably led to rearrangements of wheat homoeologue groups 6 and 7, and this also disrupted the collinearity between wheat group 6 chromosomes and rice chromosome 2. The amplification products of *TaTEF-7A* produced by three pairs of genome-specific primers were mapped on chromosome 7A. This confirmed the above opinion and indicated that grass genomes are labile, rapidly evolving entities with complex structural and functional relationships (Quraishi *et al.*, 2011).

### Advantages of genetic analysis using natural populations and NILs

Dissecting the genetics underlying complex traits in crops not only provides insights into genetic pathways, but also provides targets for MAS in breeding (Clark, 2010). Recently, candidate gene-based association analysis has been used to trace the origin of agronomically important alleles and to explore the process of domestication of cultivated rice. This approach takes advantage of historical and evolutionary recombination events in natural populations to resolve complex trait variations in individual nucleotides (Lu *et al.*, 2012). Combining linkage analysis and candidate gene association mapping, Zhang *et al.* (2014) identified a soybean gene related to P efficiency. Moreover, for pleiotropic genes, association mapping also dissects trait correlations at the gene level because polymorphic sites can be independently associated with different traits (Chen and Lubberstedt, 2010). For example, the maize pleiotropic gene *Dwarf8*, which affects both flowering time and plant height, was shown to contain two SNPs that were independently associated with each trait (Thornsberry *et al.*, 2001). The MCC contains 1% of the basic wheat germplasm collection with an estimated 70% representation of the genetic variation in that collection, making it a suitable population for detection of major QTLs controlling yield traits (Hao *et al.*, 2011; Zhang *et al.*, 2012). In this study, candidate gene association mapping showed that *TaTEF-7A* was significantly associated with yield traits. The location of this gene was consistent with the location of a previously identified QTL associated with yield and also likely to possess pleiotropic influences on grain yield traits.

NILs are a well known useful and reliable tool to evaluate trait-related allelic effects, but there are few reports of yield traits being validated by NILs in wheat (Lei *et al.*, 2012). In this study, a NIL derived from a backcross of Lumai 14 (recurrent parent, genetic background reconstitution, 93.8%) and Jinmai 47 (donor parent) was used for functional analysis of *TaTEF-7A* by comparing haplotype *Hap-7A-2* with *Hap-7A-3*. Haplotype *Hap-7A-3* had a higher GN and longer penultimate internode (Fig. 8). Hence, this result with NILs was consistent with the association analysis using natural populations and led to the conclusion that *TaTEF-7A* was the major genetic determinant controlling yield-related traits on chromosome 7A, especially grain number, which was verified by overexpression of the gene in *Arabidopsis*.

### TEF1 affects plant vegetative and reproductive development

Over the past few years, it has become apparent that TEFs regulate various aspects of plant development expressed as changes in ﬂowering, branching, leaf venation patterns, root growth, and seed development (Guo *et al.*, 2002; Grasser *et al.*, 2009; Lolas *et al.* 2010). In rice, Paul *et al*. (2010) reported a T-DNA insertion mutant of *OsTEF1*, an orthologue of *TaTEF-7A*. Functional loss of this gene led to 60–80% reduced tillering, retarded growth of seminal roots, and sensitivity to salt stress compared with wild-type Basmati 370. Detailed transcriptional profiling of *OsTEF1* revealed that mutation in the transcribed elongation factor influenced >100 genes with known function. It finely regulated tillering in rice by induction of expression of *MAX1*, a member of the cytochrome P450 family. P450s regulates various aspects of biological development, such as cell proliferation (CYP2D6) (Sandee and Miller, 2011), endosperm growth (CYP78A) (Schyman *et al.*, 2010), and grain yield-related parameters (CYP714D1) (Yang *et al.*, 2013). A pathway was proposed for TEF regulation traits through interaction with the *Cdc73* component of the *Paf1* complex in plants (Kubinski *et al.*, 2006). By its methyltransferase action on histone, the *Paf1* complex silences the expression of many genes, such as *MAX1* members (Paul *et al*., 2010). *MAX1* acts downstream of *HTD1/MAX3* and *D3/MAX4* to produce carotenoid-derived branch-inhibiting hormones, such as strigolactone and auxins (Grasser *et al.*, 2009). In this study, overexpression of *TaTEF-7A* in *A thaliana* led to pleiotropic effects affecting vegetative development, number and lengths of siliques, and grain length compared with the wild type. Clearly, this gene needs further investigation. It was also shown that *TaTEF-7A* had a significant influence on wheat yield traits by association analysis; specific haplotypes of this gene were significantly associated with GN, an important component of grain yield. This effect was confirmed in a study of NILs, and the findings indicated that *TaTEF-7A* probably has pleiotropic effects on plant development, especially on young developing spikes and seed.

### Potential application of a functional marker for *TaTEF-7A* in global wheat breeding for high GN

GN is an important yield trait that continues to attract the attention of wheat breeders. Genes contributing to high GN should be targets for selection in breeding. A significant difference in GN between *Hap-7A-3* and other haplotypes indicated that it has been a target of global wheat breeding, especially in China (Figs 11, 12). Since 1950 wheat varieties have changed 4–6 times in China, with ~10% yield increases in each cycle (Zhuang, 2003). Yield increases in China have largely depended on higher TKW and GN (Zhang *et al.*, 2012). As shown in Fig. 11, further increases in the frequency of *Hap-7A-3* should lead to higher overall grain yields. Plant breeding through phenotypic selection has made considerable progress in this regard, but it is a time-consuming and relatively inefﬁcient process (Gedye *et al.*, 2012). Recently, MAS has provided a strategy for accelerating progress. Functional markers have been developed for plant height (Wu *et al.*, 2011), vernalization response (Yan *et al.*, 2004), photoperiod response (Beales *et al.*, 2007), TKW (Jiang *et al.*, 2011; Su *et al.*, 2011), disease resistance (Gennaro *et al.*, 2009), and grain quality (Wang *et al.*, 2009). In this study, after functional verification in transgenic *Arabidopsis* of the yield-related gene *TaTEF-7A*, molecular markers based on variation in the promoter region of *TaTEF-7A* were developed to identify haplotype *Hap-7A-3*. Geographic distributions of *Hap-7A-3* indicated that such a marker for increasing GN should have worldwide application.

## Supplementary data

Supplementary data are available at *JXB* online.

Figure S1. Sequence characterization of *TEF* genes in wheat and other plant species.

Figure S2. Gene sequence of *TaTEF*.

Figure S3. cDNA sequences of *TaTEF* homoeologues.

Figure S4. PCR ampliﬁcation of cDNA in nullisomic–tetrasomic lines of homoeologous group 7.

Figure S5. Linkage disequilibrium matrix among pairwise polymorphisms in the promoter region of *TaTEF-7A*.

Table S1. The 48 cultivars used for identification of haplotypes.

Supplementary Data

## Abbreviations:

DH: double haploid
ETN: effective tiller number
GN: grain number per spike
GUS: β-glucuronidase
HD: heading date
KL: kernel length
KT: kernel thickness
KW: kernel width
LUC: luciferase
MAS: marker-assisted selection
MCC: mini core collection
MD: maturity date
NIL: near-isogenic line
PH: plant height
QTL: quantitative trait locus
SL: spike length
SN: spikelet number per spike
SNP: single nucleotide polymorphism
TEF: transcript elongation factor
TKW: 1000-kernel weight.
