# Supplementary material for: TEF-7A, a transcript elongation factor gene, influences yield-related traits in bread wheat (Triticum aestivum L.)
Source: J Exp Bot. 2014 Jul 23;65(18):5351–65. doi: 10.1093/jxb/eru306 (PMC4157721; doi:10.1093/jxb/eru306)
Supplement: Supplementary Data [file supp_65_18_5351__index.html]

 TEF-7A, a transcript elongation factor gene, influences yield-related traits in bread wheat (Triticum aestivum L.) — TEF-7A, a transcript elongation factor gene, influences yield-related traits in bread wheat (Triticum aestivum L.) — Supplementary Data 

# *TEF-7A*, a transcript elongation factor gene, influences yield-related traits in bread wheat (*Triticum aestivum* L.)

## Supplementary Data

Data files

**Files in this Data Supplement:**

- Supplementary Data - Supplementary Data
