# Supplementary material for: TEF-7A, a transcript elongation factor gene, influences yield-related traits in bread wheat (Triticum aestivum L.)
Source: J Exp Bot. 2014 Jul 23;65(18):5351–65. doi: 10.1093/jxb/eru306 (PMC4157721; doi:10.1093/jxb/eru306)
Supplement: Supplementary Data [file supp_eru306_jexbot124495_file001.pdf]

Table S1 Primer sequences used in this study

Table S2 Putative *TaTEF* orthologs in 10 plant species and yeast

Table S3 48 Cultivars used for identification of haplotypes

Fig. S1 Sequence characterization of *TEF* genes in wheat and other plant species. A, Gene structure. Boxes represent exons and lines represent introns; B, Multiple sequence alignment of domains; C, A neighbor-joining tree created with full-length protein sequences using the program MEGA5.05.

Fig. S2 Gene sequence of *TaTEF*.

Fig. S3 cDNA sequence of *TaTEF*.

Fig. S4 PCR amplification of cDNA in nullisomic-tetrasomic lines of homoeologous group 7. M, marker; 1, N7AT7B; 2, N7AT7D; 3, N7BT7A; 4, N7BT7D; 5, N7DT7B; 6, N7DT7A; 7, H_2O.

Fig. S5 Linkage disequilibrium matrix among pairwise polymorphisms in the promoter region of *TaTEF-7A*. Different colors represent different levels of LD. The labels on the x-axis are in accordance with the SNP on the y-axis in the same order.

Table S1

Primer set	Primer sequence (5'–3')		Amplified target
TaTEF-1	Forward	TGGTTACAAGTTTTGGTTGCTTC	A genome-specific
	Reverse	CAATACCAGAAACATGTAGGTCAG	
TaTEF-2	Forward	ACCTTCACACGTTTTCCTGTTC	A genome-specific
	Reverse	AACATACCAACCACAGGCTTGAG	
TaTEF-3	Forward	AGTTGCGGCTGCGCTCTT	A genome-specific
	Reverse	GCACGCCATTTTCTTCTAGA	
TaTEF-4	Forward	TGAGAATTCGACGAGAACGA	2nd time PCR for marker development
	Reverse	AGTTGCGGCTGCGCTCTT	
TaTEF-5	Forward	ATGCACCATCATCATCTTCGAC	Amplified gene and CDS sequence of TaTEF
	Reverse	GAACCATGGGGAAGAGAAAGTC	
TaTEF-6	Forward	CATCATTTGATGGATTATAACAAT	TaTEF-7A real-time PCR
	Reverse	TGCTCCCATGATTGCAGAA	
TaTEF-7	Forward	TAAGCCACCACCTAGGAAGA	TaTEF-7B real-time PCR
	Reverse	CCTGGCAGATTTGACAGTTA	
TaTEF-8	Forward	CATCATTTGATTGATTATAACCAC	TaTEF-7D real-time PCR
	Reverse	TGCTGCCATGATTGCAGAA	
Ta-GUS	Forward	CGCGGATCCAGTTGCGGCTGCGCTCTTGA	Amplification of promoter fragments
	Reverse	CCTAGGGGTTCAACTTCTTTTCTTCCTTCAA	
GAPDHF	Forward	TTAGACTTGCGAAGCCAGCA	Endogenous reference for real-time PCR
	Reverse	AAATGCCCTTGAGGTTTCCC	
At actin	Forward	CCAACAGAGAGAAGATGACT	Endogenous references for RT-PCR
	Reverse	ATGTCTCTTACAATTTCCCG	
AtTEF	Forward	GATAAGCTTGACACAATCTTTAGTT	AtTEF RT-PCR
	Reverse	TCAGAAGTTATACTTCCTTTTGACA	
TaTEF	Forward	AGAGAAAGTCAGCTGCTAAGC	TaTEF RT-PCR
	Reverse	ACTCATCGATCCATTCGCT	

Table S2

Species	Classification	Gene name	Accession no.
Brachypodium distachyon	Monocot	*BdTEF*	Bradi1g49230
Oryza sativa	Monocot	*OsTEF*	LOC_Os02g04160
Setaria italica	Monocot	*SiTEF*	Si007649m.g
Zea Mays	Monocot	*ZmTEF*	GRMZM5G858444
Sorghum bicolor	Monocot	*SbTEF*	Sb10g003710
Arabidopsis thaliana	eudicot	*AtTEF*	AT5G46030.1
Thellungiella halophila	Eudicot	*ThTEF*	Thhalv10001045m.g
Brassica rapa	Eudicot	*BrTEF*	Bra022011
Citrus sinensis	Eudicot	*CsTEF*	orange1.1g034538m.g
Physcomitrella patens	Eudicot	*PpTEF*	Pp1s75_70V6
Saccharomyces cerevisiae	Funguis (yeast)	*ByTEF*	NC_001143

Table S3

Accession	Cultivars	Genotype
SJZ8	Shijiazhuang 8	Hap-7A-3
YZ1	Yanzhan 1	Hap-7A-3
S4185	Shi 4185	Hap-7A-3
YM18	Yumai 18	Hap-7A-3
WM6	Wenmai 6	Hap-7A-3
JM47	Jinmai 47	Hap-7A-3
SN229	Shannong 229	Hap-7A-3
JM8	Jinmai8	Hap-7A-3
SM3	Sumai3	Hap-7A-3
XKH9	Xinkehan9	Hap-7A-3
ZY4	Zhengyin4	Hap-7A-3
GS96	Gansu96	Hap-7A-3
NY188	Neixiang 188	Hap-7A-2
BN6	Beinong 6	Hap-7A-2
AM6	AM6	Hap-7A-2
PY27	Pingyang 27	Hap-7A-2
DFH3	Dongfanghong3	Hap-7A-2
GT	Guangtou	Hap-7A-2
ZM9023	Zhengmai 9023	Hap-7A-1
JXZ	Jiangxizao	Hap-7A-1
ND2419	Nanda 2419	Hap-7A-1
YD1817	Yanda 1817	Hap-7A-1
LM14	Lumai14	Hap-7A-1
QCM	Qiangchangmai	Hap-7A-1
WSB	Wangshuibai	Hap-7A-1
NQ4	Ningchun 4	Hap-7A-1
J411	Jing 411	Hap-7A-1
CS	Chinese Spring	Hap-7A-1
LZ953	Laizhou 953	Hap-7A-1
BMZ	Baimaizi	Hap-7A-1
ZY9507	Zhongyou 9507	Hap-7A-1
DBP	Dabaipi	Hap-7A-1
XHP	Xiaohongpi	Hap-7A-1
DXZ	Dingxingzhai	Hap-7A-1
LHM	Lanhuamai	Hap-7A-1
DMHM	Daimanghongmai	Hap-7A-1
ZLDM	Ziludongmai	Hap-7A-1
FK2	Fengkang2	Hap-7A-1
QM	Qianmai	Hap-7A-1
HSB	Huangshuibai	Hap-7A-1
BP	Baipu	Hap-7A-1
ZXM	Zaoxiaomai	Hap-7A-1

HD6	Huangdong6	Hap-7A-1
LY5	Liying5	Hap-7A-1
DQM	Daqingmang	Hap-7A-1
OF	Orofen	Hap-7A-1
AT	Atlas 66	Hap-7A-1
CAXM	Chaoanxiaomai	Hap-7A-1

Fig. S1

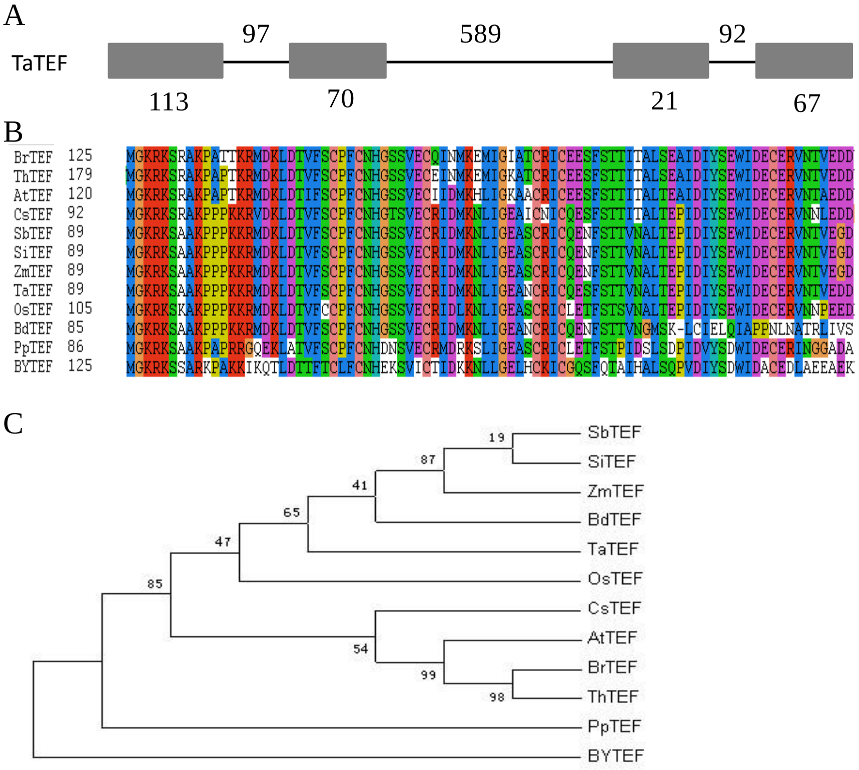
A
TaTEF
97
589
92
113
70
21
67
B
BrTEF 125
ThTEF 179
AtTEF 120
CsTEF 92
SbTEF 89
SiTEF 89
ZmTEF 89
TaTEF 89
OsTEF 105
BdTEF 85
PpTEF 86
BYTEF 125
C
19
87
41
65
47
85
54
99
98
SbTEF
SiTEF
ZmTEF
BdTEF
TaTEF
OsTEF
CsTEF
AtTEF
BrTEF
ThTEF
PpTEF
BYTEF

Fig. S2

```
                10        20        30        40        50        60        70        80        90        100       110       120       130       140       150
1     ATGCACCATCATCATCTTCGACAGTGTTGACGCGCTCGCACTCATCGATCCATTCGCTGTATCTGCAGGAAAATTAATATGATCTTCAGTTCCTGGTCTC--TGACGTATTTGCACCAACTCACTAGTCAAGAGTTATTTGTTGAATTTG TaTEF-A
1     ATGCACCATCATCATCTTCGACAGTGTTGACGCGCTCGCACTCATCGATCCATTCGCTGTATCTGCAGGAAAGATAATACGATCTTCAGTTCCTGGTCTCCTTGACGTATTTGAACCAACTCACTAGTCAAGAGTTATTTGTTGGATTTG TaTEF-B
1     ATGCACCATCATCATCTTCGACAGTGTTGACGCGCTCGCACTCATCGATCCATTCGCTGTATCTGCAGGAAAGATGATACGATCTTCAGTTCCTGGTCTC--TGACGTATTTGCACCAACTCACTAGTCAAGAGTTATTTGTTGGATTTG TaTEF-D

                160       170       180       190       200       210       220       230       240       250       260       270       280       290       300
149   ACTTACATATCAATAGGCTCAGTCAGCGCTGCACCAAAGAAAACAGAGAATTAGTACAAT---------ACCAGAAACATGTAGGTCAGCAAGGGTA-AAAAATTCTACTGAACACTGTTTCCTTTACATGAGATAG--------TTATA TaTEF-A
151   ACTTACATATCAATAGGCTCAGTCAGCGCTGCACCAAAGAAAACAGAGAATTAGTACAAT---------AGCAAAAACATGCAGCTCAGCAAGGGTACAAAAACTCTACTGAACA-TGTTTCCTTTACATGACATAG--------TTATA TaTEF-B
149   ACTTACATATCAATAGGCTCAGTCAGCGCTGCACCAAATAAAACAGATAATTAGTACAATATACAATATACTAAATACATGTAGCTTAGCAAGGGTA-AAAAACTCTACTGAACACTGTTTCCTTTACATGACATGGTTATAGTGTTATA TaTEF-D

                310       320       330       340       350       360       370       380       390       400       410       420       430       440       450
281   CACAAACTTGAAAACATGTGGCACAACAATGGTTAACCCTCTACAACACTGTTTTTCATTTAAGCAATATTATTATACAGGTTCAGTTCTGAACTTGTGATGATGCAGGTTTGTTTCAAGCAATTCAGTTAACTCAGACTTGGA-CACGA TaTEF-A
283   CACAAACTTGAAAACATGTGGCACAACAAAGGTTAACCCTCTACAACACTGTTTT-CATTTAAGCAATATTAGTATGCAGGTTCAGTTCTAAACTTATGATGATGCAGGTTTGTTTCAGGCAATTCAGTTAACTCAGACTTGGAACATGA TaTEF-B
298   CACAAACTTGAAAACATGTGGCACAACAAAGGTTAACCCTCTACAACACTGTTTTTCATTTAAGCAATACTATTATACAGGTTCAGTTCTGAACTTCTGACGATGCAGGTTTATTTCAAGCAATTCAGTTAACTCAGACTTGGAACATGA TaTEF-D

                460       470       480       490       500       510       520       530       540       550       560       570       580       590       600
430   TAAACATAGGGAGCCACATGACCTGCCTTACAGACATGATGGTGTGATT-AATGGCAACCAGGTAAGAATATAACTGTACATGATTGCTACCTCGGCTCTGCTTCATTATGACCTAGTTGATTAGAATGACTAAACCCCCAA-GAGTATA TaTEF-A
432   AAAA-AAAGGGAGCCACATGGCCTATCTTACAAGCATGGTTGTGTGATTTAATGGCAACCAAGTACGCATATAACCGTACATCATTACTACTTCGGCTCTGCTCCATCATGACCTAGTTGATTAGAATGACTAAA----------GTATA TaTEF-B
448   AAAA-ATAGGGAGCCACAGGGCTTATCTTACAAGCATGGTTGTGTGATTCAATGACAACCAAGTACGCATATAACTGTACAAGATAACTACTTCGGCACTGCTCCATTATGACCTAGTTGATTAGAATGACTAAAACCCTAAAGAGTATA TaTEF-D

                610       620       630       640       650       660       670       680       690       700       710       720       730       740       750
578   TGAGATGGACATGGCTGTAGGAAATTTATAGATA--AGCTATACCCTGTTCTTAAAGCTGTAAGCATTAGTTTGAGAAGAAACAAAAACAATTGAGATGTCTAGAGCCAAATTT-CTAGCTGAGAATCAATCAGTACAA-CATCTGCATT TaTEF-A
571   TGAGATAGGCATGGCTGTAGGAAAGTTTTAGATATAAGCTATACCATGTTTCTAGAGCTGCAAGCATTAGGTTGAGAAGAAACAAAAACAAT-GAGATGTCTGGTGCGGGGTTTCCTAGCTGAGAATCAATCAGTATGGGCATCTACTTT TaTEF-B
597   TGAGATAGGCATGGTTGCAGGAAATTTATAGATA--AGCTATACCATGTTCCTAGAGCTGTAAGCATTAGGTTGAGAAGAAACAAAAACAAT-GAGATGTCTGGTGCGGAATTTTCTAGCTGAGAATCAATCAGTATGGGCACCTACTTT TaTEF-D

                760       770       780       790       800       810       820       830       840       850       860       870       880       890       900
724   AGGAGGTAAAATCTGCAGTTTTATGTAAATTTTGGGCATACCGTTTACGGTTGTGCTGAAGCTTTCCTGGCAGATTCGACAATTTGCCTCACCAATCAGATTCTTCATATCACTGAAG---AAAAAAAGGGGGAAACCCCAGTCACAATG TaTEF-A
720   AGGAG-TACAATTTGCAGT--TATGTAAATTTTGGGCATACCGTTTACGGTTGTGCTGAAGCTTTCCTGGCAGATTCGACAATTTGCCTCACCAATCAGATTCTTCATATCACTGAAG---AAAAAAAGGGGGAAACCCCAGTCACAATG TaTEF-B
744   AGGAG-TACAATTTGCAGTTCTATGTACAAATTGGACATACCATTTACAGTGGTGCTGAAGCTTTCCTGGCAGATTCGACAATTGGCCTCACCAATCAGATTCTTCATATCACTGAAGGGAAAAAAAAGAGGGAAACCCCAGTCACAATG TaTEF-D

                910       920       930       940       950       960       970       980       990       1000      1010      1020      1030      1040      1050
871   CTGAATTGATAGCCATCCAATATCAACCAATG-AACAGGAAA-CGTGTGAAGGTAGTACTAACATTCGGCATTCAACACTGCTCCCGTGATTGCAGAACGGGCAGGAAAAGACCGTGTCGAGCTTGTCCATCCGCTTCTTAGGGGGTGGC TaTEF-A
864   CTGAATTGATAGCCATCCAATATCAACCAATG-AACAGGAAA-CGTGTGAAGGTAGTACTAACATTCGGCATTCAACACTGCTCCCATGATTGCAGAACGGGCAGGAAAAGACCGTGTCGAGCTTGTCCATCCGCTTCTTAGGGGGTGGC TaTEF-B
893   TTGAATGGATAGCAATCCAGTACCAACCAATTTAACAGGAAAACATGTGAAGACAGTACTAACATTCGGCATTCAACACTGCTGCCATGATTGCAGAACGGGCAGGAAAAGACAGTGTCGAGCTTGTCCATCCGCTTCTTAGGAGGTGGC TaTEF-D

                1060      1070      1080
1019  TTAGCAGCTGACTTTCTCTTCCCCATGGTTC TaTEF-A
1012  TTAGCAGCTGACTTTCTCTTCCCCATGGTTC TaTEF-B
1043  TTAGCAGCTGACTTTCTCTTCCCCATGGTTC TaTEF-D
```

Fig. S3

```
         10        20        30        40        50        60        70        80        90
ATGGGGAAGAGAAAGTCAGCTGCTAAGCCACCCCCTAAGAAGCGGATGGACAAGCTCGACACGGTCTTTTCCTGCCCGTTCTGCAATCAT A
ATGGGGAAGAGAAAGTCGGCTGCTAAGCCACCACCTAGGAAGAGGATGGACAAGCTCGATACCGTCTTCTCCTGCCCATTCTGCAACCAT B
ATGGGGAAGAGAAAGTCAGCTGCTAAGCCACCTCCTAAGAAGCGGATGGACAAGCTCGACACTGTCTTTTCCTGCCCGTTCTGCAATCAT D

        100       110       120       130       140       150       160       170       180
GGGAGCAGTGTTGAATGCCGAATTGATATGAAGAATCTGATTGGTGAGGCAAATTGTCGAATCTGCCAGGAAAGCTTCAGCACAACCGTA A
GGGAGCAGCGTTGAGTGCCGGATTGATTTGAAGAATCTGATTGGTGAGGCTAACTGTCAAATCTGCCAGGAAAGCTTCAGCACCACTGCA B
GGCAGCAGTGTTGAATGCCGAATTGATATGAAGAATCTGATTGGTGAGGCCAATTGTCGAATCTGCCAGGAAAGCTTCAGCACCACTGTA D

        190       200       210       220       230       240       250       260       270
AACGCGCTGACTGAGCCTATTGATATATACAGCGAATGGATCGATGAGTGCGAGCGCGTCAACACTGTCGAAGATGATGATGGTGCATGA A
AATGCGCTGACTGAACCTATTGATGTATACAGCGAATGGATCGATGAGTGCGAGCGCGTCAACACTGTCGAAGATGATGATGGTGCATGA B
AATGCGCTGACTGAGCCTATTGATATATACAGCGAATGGATCGATGAGTGCGAGCGCGTCAACACTGTCGAAGATGATGATGGTGCATGA D
```

Fig. S4

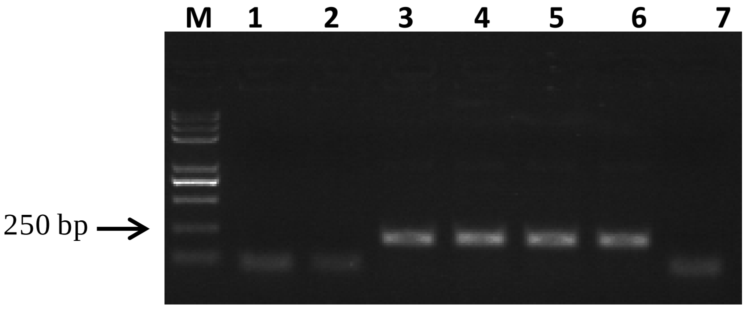

Fig. S5

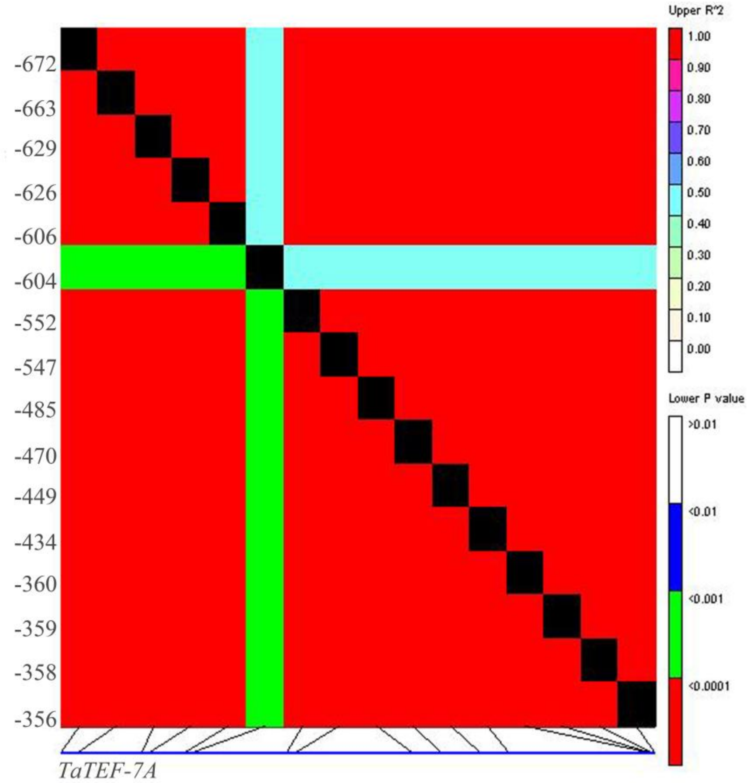
-672
-663
-629
-626
-606
-604
-552
-547
-485
-470
-449
-434
-360
-359
-358
-356
TaTEF-7A
Upper R^2
1.00
0.90
0.80
0.70
0.60
0.50
0.40
0.30
0.20
0.10
0.00
Lower P value
>0.01
<0.01
<0.001
<0.0001
